# Failure of a patient-centered intervention to substantially increase the identification and referral for-treatment of ambulatory emergency department patients with occult psychiatric conditions: a randomized trial [ISRCTN61514736]

**DOI:** 10.1186/1471-227X-5-2

**Published:** 2005-05-09

**Authors:** David L Schriger, Patrick S Gibbons, Wais A Nezami, Carol A Langone

**Affiliations:** 1 University of California, Los Angeles Emergency Medicine Center, Los Angeles, CA, USA; 2 University of California, Los Angeles School of Medicine, Los Angeles, CA, USA; 3 Department of Clinical Social Work, University of California, Los Angeles Medical Center, Los Angeles, CA, USA

## Abstract

**Background:**

We previously demonstrated that a computerized psychiatric screening interview (the PRIME-MD) can be used in the Emergency Department (ED) waiting room to identify patients with mental illness. In that trial, however, informing the ED physician of the PRIME-MD results did not increase the frequency of psychiatric diagnosis, consultation or referral. We conducted this study to determine whether telling the patient and physician the PRIME-MD result would result in the majority of PRIME-MD-diagnosed patients being directed toward treatment for their mental illness.

**Methods:**

In this single-site RCT, consenting patients with non-specific somatic chief complaints (e.g., fatigue, back pain, etc.) completed the computerized PRIME-MD in the waiting room and were randomly assigned to one of three groups: patient and physician told PRIME-MD results, patient told PRIME-MD results, and neither told PRIME-MD results.

The main outcome measure was the percentage of patients with a PRIME-MD diagnosis who received a psychiatric consultation or referral from the ED.

**Results:**

183 (5% of all ED patients) were approached. 123 eligible patients consented to participate, completed the PRIME-MD and were randomized. 95 patients had outcomes recorded. 51 (54%) had a PRIME-MD diagnosis and 8 (16%) of them were given a psychiatric consultation or referral in the ED. While the frequency of consultation or referral increased as the intervention's intensity increased (tell neither = 11% (1/9), tell patient 15% (3/20), tell patient and physician 18% (4/22)), no group came close to the 50% threshold we sought. For this reason, we stopped the trial after an interim analysis.

**Conclusion:**

Patients willingly completed the PRIME-MD and 54% had a PRIME-MD diagnosis. Unfortunately, at our institution, informing the patient (and physician) of the PRIME-MD results infrequently led to the patient being directed toward care for their psychiatric condition.

## Background

There is a higher prevalence of psychiatric conditions in patients presenting to emergency departments (ED)s with non-emergent complaints than in the general population [1-4]. On average, patients with untreated psychiatric illness have more frequent ED visits and use more health care services than those in the general public [5,6]. Studies in a variety of ED settings have documented that over 40% of ambulatory patients have underlying psychiatric conditions [1-4]. Since there are highly effective treatments for many psychiatric conditions, these patients are generally expected to achieve better health outcomes if their psychiatric condition is diagnosed and treated, especially when their somatic complaints (weak and dizzy, back pain, etc.) are not amenable to effective treatment. A collateral benefit would be a reduction in societal health care costs [7].

PRIME-MD is a screening tool for psychiatric conditions that uses closed ended questions to make DSM-IV diagnoses [8]. Its diagnostic validity has been established in a number of ambulatory care settings [9-12]. We have demonstrated that the computer version of the PRIME-MD can be used in the ED waiting room to identify patients with underlying psychiatric conditions that might be causing or exacerbating their somatic presenting complaints [4]. Unfortunately, in that study, the emergency physicians ignored the patient's PRIME-MD diagnoses and neither diagnosed nor treated the patient's mental health disorder. The same phenomenon has been observed in primary care [13].

In this study, we conducted focus groups to determine why our first trial failed and then conducted a trial using the stronger intervention of informing both the patient and the physician of the computer's findings. Our goal was to determine whether this intervention would result in the majority of patients with a PRIME-MD diagnosis being referred for evaluation and treatment of their psychiatric condition. By empowering the patient to act as his or her own advocate, we hoped to overcome whatever factors deter physicians from exploring these diagnostic possibilities with the patient. We designed a 3-limb randomized trial that included a control group, a group in which only the patient was informed of the PRIME-MD results and a group in which both patient and physician were informed of the results.

## Methods

### Study design and setting

Focus groups – An experienced facilitator used a set of open ended probes to conduct two 90 minute focus groups to explore why physicians might be reluctant to pursue psychiatric diagnosis, consultation or referral in patients given a psychiatric diagnosis by PRIME-MD. One group included 6 randomly selected EM residents, the other 6 EM faculty. Two observers took notes and identified main themes and points of disagreement. The results informed the development of the intervention and study materials.

Trial – This randomized, controlled clinical trial was conducted at the University of California Los Angeles Emergency Department, a teaching hospital and Level I trauma center. The annual census is 44,000. Study subjects included emergency and internal medicine house staff, emergency medicine faculty, and enrolled patients, all of whom were consented. The study was approved by the UCLA IRB.

### Selection of participants

Patients age 18 or older presenting to the ED between the hours of 10 a.m. and 9 p.m. on most (84%) weekdays from March to September 2002 were recruited for study participation. A trained research assistant, stationed at the triage desk, listened to each intake interview and identified adults with diffuse somatic complaints (e.g., vague head, abdominal, back or body pain of non-acute onset; generalized weakness; "don't feel well") that did not seem to mandate emergency care or did not coincide with physical findings (e.g. complaining of rash but no rash visible). Patients arriving via ambulance, patients in extremis, and patients who indicated they were not comfortable reading English were excluded; as were patients whose symptoms suggested psychiatric illness (e.g. hearing voices, suicidal ideation, anxiety...), patients with recent substance abuse, and patients who already participated in the trial.

### Intervention

Consenting patients were asked to complete the self-administered, PRIME-MD computerized psychiatric questionnaire (version 1.2, Pfizer, Inc, New York, NY) in the ED waiting room prior to seeing the physician. PRIME-MD poses a series of questions to screen patients in 7 psychiatric domains (mood, anxiety, alcohol abuse, eating disorder, obsessive-compulsive disorder, phobia, and somatization). Positive responses to these screening questions trigger additional questions to confirm or reject particular diagnoses within each domain. When the session is complete, the program prints a grid that indicates the presence or absence of 20 specific diagnoses within the 7 domains.

Patients were seated at a computer secluded from the rest of the waiting room. They answered questions using a Fastpoint light pen (Fastpoint Technologies, Stanton, CA). A research associate was present to assist with technical issues, time the session, and record any difficulties with the hardware or software. Upon conclusion of the PRIME-MD interview, but before results were known, the randomization software, using the random number function in STATA 6.0, assigned the patient to one of three groups: results given to patient and doctor (40%), results given only to patient (40%), or results given to neither (20%). Patients in the first two groups were given a packet with their PRIME-MD results, an explanation of these results, a glossary of terms, and a cover letter encouraging them to share their PRIME-MD results with the treating ED physician. All patients, regardless of randomization group, were provided a written invitation to speak with a psychiatric social worker at the conclusion of their ED visit All patients with PRIME-MD diagnoses "major depression" or "r/o bipolar disorder" were assessed for suicidality by the psychiatric social worker prior to discharge.

The physicians caring for patients in the "tell both" group were provided PRIME-MD results through identical pre-printed Post-it™ notes affixed over the parts of the medical record where the resident and the attending write their notes. Each Post-it™ note indicated which PRIME-MD domains were positive (or that the patient had no PRIME-MD diagnoses) and referred the physician to additional materials attached to the chart. These materials included a one-page cover sheet that introduced the physician to PRIME-MD, reviewed the evidence of its validity, and listed the patient's PRIME-MD diagnoses. As part of the study we produced a list of low-cost and no-cost psychiatric care options that could be offered to patients as part of their after care instructions. Unfortunately, many of the agencies on the list have many-month-long waiting lists for patients who do not have an acute psychiatric issue. Apart from these interventions, there was no attempt to alter usual care.

### Methods of measurement

The primary outcome was whether each patient diagnosed by the PRIME-MD software left the ED having had a psychiatric consultation or with a referral for further evaluation and treatment of their psychiatric condition. Any indication that the patient was referred to a health care provider for help with a mental health issue, including a statement that the low-cost no-cost sheet had been provided was considered evidence of referral. Secondary outcomes were: whether the ED physician made a psychiatric diagnosis and whether the patient actually received follow-up for the psychiatric condition. This information was gleaned from standardized review of the medical record and after care instructions, and follow-up patient telephone interview which took place 2 to 4 weeks after the visit. We questioned the treating resident just after the patient had left the ED to ascertain whether she was aware of the PRIME-MD results and how she had acted upon them. Patient and physician interview procedures and results can be found in the Appendix.

### Data collection and processing

Two investigators independently reviewed the chief complaints of all enrolled patients (presented independently of all other data) and excluded patients who had been inappropriately entered into the study. Trained research associates abstracted the patient's demographics, chief complaint, diagnoses, psychiatric consultations and psychiatric referrals from the ED medical record to a standardized form. Interrater reliability was assessed on a 10% sample of charts. Abstractor's were blinded to the patients' randomization status and PRIME-MD results. STATA 8.0 was used for data verification, database management, and statistical analysis. To maintain anonymity and confidentiality, physician and patient identifiers were dropped during database creation and replaced with randomly assigned numbers.

### Primary data analysis

We designed this study to estimate the treatment effect with reasonable precision, not to perform formal hypothesis tests [14]. We took a Bayesian approach to this trial and incorporated data from our previous study and other reasonable priors in the analysis [15]. We used beta distributions to model all priors and likelihoods. For the control limb we set a prior distribution of beta (1.2, 15.8) which has a mean of 7% and a 95% credible interval of 0% to 23%. This prior is based on the control limb or our previous trial (7% (3 of 45) successes) but was widened to account for potential differences between the trials. It's information content is equivalent to a 17 person study. For the two other limbs we used beta (0.3, 1.2) distributions which have a mean of 20% and 95% credible interval of 0% to 87%. This wide interval reflected our uncertainty regarding the effect of the intervention and has information content equivalent to a study with a sample size of 1.5 subjects. All Bayesian calculations were performed in FAST*PRO and STATA 8.0 [16].

Our determination of sample size was guided by Bayesian estimations and traditional frequentist calculations. We decided a priori that the intervention was only worth doing if it resulted in more than 50% of subjects with a PRIME-MD diagnosis being offered referral. Our reasoning was that given the myriad of competing demands in the ED setting this particular intervention had to have a substantial impact (not just an incremental improvement over the status quo) in order to justify the cost and effort expended. As mentioned above, the control group of our previous trial had 3 of 45 patients with a PRIME-MD diagnosis receive psychiatric consultation or referral. A frequentist two limb trial would require 21 patients per group (80% power for difference between 50% and 7%, alpha .05). We sought to behav 80 patients in each active limb since we conservatively expected that at least 1/3^rd ^of enrolled subjects would have a PRIME-MD diagnosis (42% did in our first study). Since the prior distribution for the control limb was considerably narrower than for the other limbs, we decided to randomize half that number of patients to this limb so that the posterior distributions of all limbs would be of similar precision.[15]. To conserve resources, we planned an interim analysis once we enrolled 40 patients in each active limb to determine whether the study had any chance of producing a clinically important result. Data analysts were blinded to the identity of the 3 experimental groups and the randomization proportions.

## Results

### Focus groups

There was general uniformity of opinion on the following themes: residents do not feel adequately trained to detect and treat occult psychiatric illness, attending physicians were concerned that focusing on psychiatric issues may cause the housestaff (but not them) to overlook somatic illness, and screening and diagnosis was pointless in a system that lacked any viable means for providing follow-up or treatment. No one questioned the validity of the PRIME-MD. There was heterogeneity of opinion regarding whether the ED was the proper place to screen and diagnose psychiatric conditions even if adequate follow-up was available. Some ED residents and attendings candidly revealed that they "did not go into emergency medicine to make non-emergent psychiatric diagnoses."

### Randomized trial

**Figure 1:**
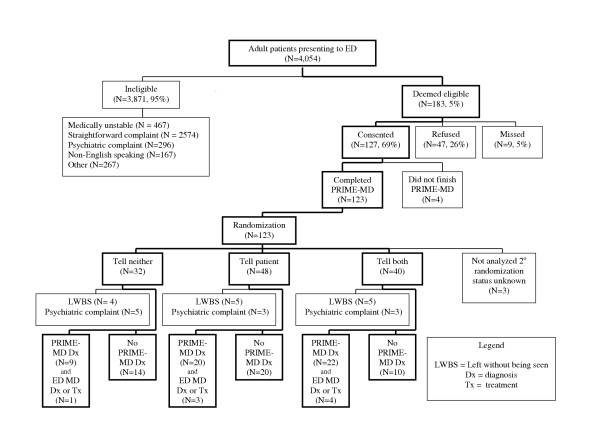
This graphic shows how the 95 subjects tallied in the final analysis were culled from the 4,054 patients seen in the ED during study hours. Patients were randomized to three groups: tell neither (PRIME-MD results not shared with the patient or physician – the control group), tell patient (PRIME-MD results shared with the patient), and tell both (PRIME-MD results shared with patient and physician). In the lowest level of boxes, the entry "ER MD Dx or Tx" indicates how many patients diagnosed by PRIME-MD were diagnosed, treated or referred by the emergency physician.

At the time of the interim analysis and the stopping of the study, 4,054 patients had been triaged. 183 were eligible and 127 were randomized (Figure 1). 14 patients left prior to being seen, 4 did not complete their PRIME-MD session, and 11 patients were excluded after randomization when it was determined they had an unappreciated psychiatric complaint (e.g. "I haven't slept in 3 nights"). 95 patients were included in the analysis: 32 in the tell-both limb, 40 in the tell-patient limb and 23 in the tell-neither control limb (Table 1). Frequent chief complaints were musculoskeletal pain or minor trauma (23%), abdominal pain +/- nausea +/- vomiting (23%), weak +/- dizzy (12%), and headache (9%). No resident or attending saw a disproportionate number of patients (Table 1).

**Table 1:** Physician and patient information by study limb

	**Study Limb**			
	**Tell Neither**	**Tell Patient**	**Tell Both**	**Total**
**Patient Characteristics**				
No. of patients	23	40	32	95
Age (y) mean (SD)	48(15)	46(14)	48(15)	47(15)
Female, No. (%)	12(53)	17(43)	19(60)	48(51)
				
**Physician Characteristics**				
No. of Attendings	12	17	15	20
No. of Residents	19	30	30	51
				
**No. of residents seeing:**				
1 patient	18	22	25	31
2 patients	1	4	3	9
3 patients	0	2	0	7
≥ 4 patients	0	0	0	4

The PRIME-MD session took between 4 and 8 minutes for half the patients (range 2–35 minutes). PRIME-MD made one or more diagnoses in 51 of the 95 (54%) patients (Table 2). Mood and anxiety disorders predominated. 24 patients were diagnosed with major depression and 4 with r/o bipolar disorder. The psychiatric social worker identified 2 patients in this group who were suicidal. The physicians of both of these patients were informed and appropriate assessment and treatment was instituted.

**Table 2:** PRIME-MD diagnoses by study limb

	**Study Limb**			
	**Tell Neither**	**Tell Patient**	**Tell both**	**Total**
No. of patients	23	40	32	95
**Patients with**				
Any PRIME-MD diagnostic domain (%)	9(39)	20(50)	22(69)	51(54)
1 PRIME-MD diagnostic domain	3(13)	8(20)	10(31)	21(22)
2 PRIME-MD diagnostic domains	2(9)	7(18)	5(16)	14(14)
3 PRIME-MD diagnostic domains	4(17)	1(3)	5(16)	10(11)
≥ 4 PRIME-MD diagnostic domains	0(0)	4(10)	2(6)	6(6)
				
Any mood diagnosis	7(30)	12(30)	17(53)	36(37)
Any anxiety diagnosis	4(17)	14(35)	10(31)	28(29)
Any OCD diagnosis	5(22)	6(15)	7(22)	18(19)
Any alcohol/dependence	1(4)	5(13)	6(19)	12(13)
Any phobia diagnosis	2(9)	5(13)	2(6)	9(9)
Any eating disorder	0(0)	0(0)	2(6)	2(2)

**Figure 2:**
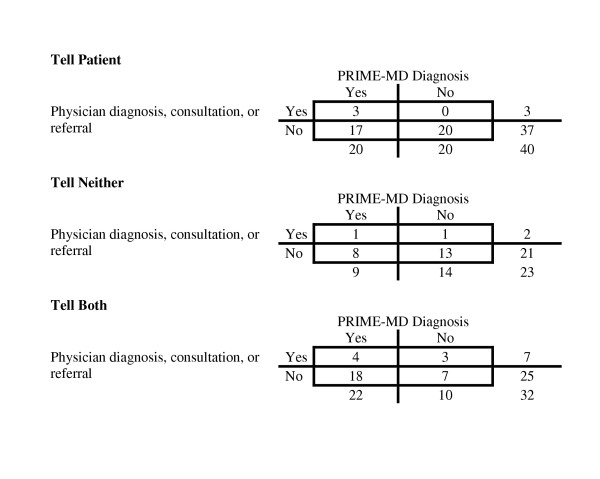
Title: PRIME-MD diagnosis versus whether the ED physician made a psychiatric diagnosis, consultation or referral, by study limb. For each study limb a 2 × 2 table depicts how each patient's PRIME-MD status (psychiatric diagnosis versus none) compares to emergency physician's decision to make a psychiatric diagnosis, obtain psychiatric consultation, or refer the patient for evaluation of the psychiatric condition.

Interrater agreement for the chart abstraction exceeded 90% for all items. 16% of patients diagnosed by the PRIME-MD were given a psychiatric consultation or referral in the ED (Table 3). While there was a suggestion that the intervention increased the likelihood that patients would be identified and offered treatment (control 11%, tell-patient 15%, tell-both 18%), no group approached the 50% level that we sought (Table 3 and Figure 2).

**Table 3:** Psychiatric diagnosis, consultation and referral in patients with a PRIME-MD diagnosis, by study limb (N (%))

	**Study Limb**		
	**Tell Neither**	**Tell Patient**	**Tell Both**
Patients with a PRIME-MD diagnosis and a:	9	20	22
Physician psychiatric diagnosis	0 (0%)	2 (10%)	4 (18%)
Physician psychiatric consultation or referral	1 (11%)	3 (15%)	4 (18%)
Physician psychiatric diagnosis, consultation, or referral	1 (11%)	3 (15%)	4 (18%)
Frequentist 95% CI	0%, 48%	3%, 38%	5%, 40%
Bayesian Analysis of these data*	mean 95% Credible Interval		
Prior distribution for limb	7% 0%, 23%	20% 0%, 87%	20% 0%, 87%
Likelihood for limb (from observed data)	11% 0%, 37%	15% 3%, 33%	18% 5%, 36%
Posterior distribution for limb	9% 1%, 22%	15% 4%,33%	18% 6%, 36%

*All probabilities are modelled as beta distributions. The traditional, frequentist 95% confidence intervals for the trial can be compared to the 95% credible intervals of the posterior distributions produced by the Bayesian analysis which combines prior beliefs about the results of each limb with the observed data (see text and [14]).

From the physician and patient questionnaires (response rates 41% and 55% respectively, see Appendix for details) we learned that: most physicians with a patient in the tell-both group learned that patients' PRIME-MD status from the stickers, not from the patient. Few patients said they told their ED physician about the PRIME-MD results and physicians confirmed that they were seldom told such information. While physicians generally agreed with the PRIME-MD diagnoses, they did not choose to act on them, most commonly because "the visit wasn't about that issue." Only 2 of 28 patients contacted at least 2 weeks after the ED visit reported seeing a physician or mental health care professional about their psychiatric problem.

## Discussion

This small trial, when interpreted in the context of our previous trial [4], is sufficient to make the following points: 1) it is feasible to use the PRIME-MD in the ED, 2) there is a high prevalence of psychiatric illness in the ambulatory ED population, 3) in our ED, when physicians or patients or both are informed of the presence of a PRIME-MD psychiatric diagnosis, few psychiatric diagnoses are made and few patients receive consultation or referral to address the problem, 4) our strategy of using the patient to initiate a discussion of the patient's mental health did not work.

While 18% (95% Credible Interval 6%, 36%) of patients in the tell-both group were given consultation or referral, 10% (95% Credible Interval -9%, 28%) more than in the control group, we do not believe that the improvement was of sufficient magnitude to justify using PRIME-MD in the waiting room of our ED.

These findings are a bit disconcerting since the treatments for many of these psychiatric conditions are highly effective and the morbidity of the conditions is considerable. Furthermore, treatments for conditions related to the patient's chief complaints (low back pain, general weakness, etc.) have limited efficacy. It would therefore be highly desirable to develop a method for identifying and treating these patients' psychiatric conditions.

The focus groups and this trial suggest that emergency physicians are reluctant to consider psychiatric illness in patients presenting with somatic complaints for reasons such as: discomfort with the content and length of the psychiatric interview, lack of knowledge of diagnostic criteria, lack of interest in the activity, and belief that the activity is futile since the health care system provides few resources for the non-psychotic, underinsured patients who are the most likely target of this intervention. It might be possible to have the computer refer patients directly to providers, thereby bypassing the physicians' resistance, but this could only occur in a system where viable referral options exist. Until such time, it is unlikely that a successful intervention can be developed until readily available follow-up becomes available.

This study took place in a U.S. academic ED within a health care system that provides extremely limited mental health follow-up for underinsured and uninsured patients who are not homicidal, suicidal or floridly psychotic, the majority of patients in our study. Results might be different in a system (such as the U.K.'s) that provides reasonable treatment options for such patients. The inclusion criteria are somewhat subjective. It is quite likely that eligible patients were missed. Furthermore, it is possible that PRIME-MD produced false positive and false negative results. Nevertheless, it is highly unlikely that these selection and misclassification biases would affect our conclusion. The small size of our study makes our estimates imprecise, but not so much so that our conclusions are jeopardized. The poor response rates for physician and patient questionnaires seriously compromises their utility.

We powered the study to look for large effects and cannot comment on whether the use of PRIME-MD produces a modest increase in psychiatric diagnosis and referral in patients with occult psychiatric illness. In retrospect, we might have listened to the focus group participants more carefully. They candidly told us why they were not keen to diagnose and treat these patients. We ignored this and designed an intervention that attempted to use the patients to force their hand. We might have had more success had we organized a follow-up clinic for such patients and directly addressed the physicians' attitudes. We also learned that while the trained undergraduate student research assistants did quite a good job identifying, consenting, and enrolling patients, they were less effective with the physician questionnaire (40% completion rate). We were unrealistic to expect that a junior person could capture the attention of a busy ED resident to get the questionnaire completed. Finally, we might have used block randomization to avoid the imbalance in assignments that occurred in this study.

## Conclusion

In summary, in our ED, telling PRIME-MD diagnoses to the patient (and physician) did not substantially increase the proportion of patients whose were diagnosed and referred for treatment of their psychiatric condition in a health care system that offered extremely limited follow-up possibilities for such patients.

## Competing interests

The study was funded in part by an unrestricted gift from the Pfizer Corporation. Apart from providing financial support, Pfizer had no involvement in the planning, conduct, analysis or reporting of the trial.

## Authors' contributions

DS and PG conceived and designed the study. PG and WN coordinated the study. CL conducted the patient interviews and performed many of the psychiatric interviews. All authors participated in the drafting and editing of the manuscript. DS takes responsibility for the conduct and reporting of the research.

## Appendix

### Methods

### Physician questionnaire

Participating residents were approached as soon as each patient was discharged from the ED, regardless of randomization status. A research assistant administered one of two versions of a structured questionnaire. Physicians whose patient had a PRIME-MD diagnosis were asked whether they knew the PRIME-MD diagnoses, how they came to know them, whether they agreed with them (and why), and whether they took any actions (and why). Physicians whose patient did not have a PRIME-MD diagnosis were asked if they knew the patient's PRIME-MD status.

### Patient questionnaire

The psychiatric social worker attempted to call each patient during a 2 week interval beginning 2 weeks after the ED visit. Each patient had been asked to provide a current phone number at the time of behaviour. Patients were asked 15 closed-ended questions about their current physical and mental health in comparison to the day of the ED visit, their knowledge of their PRIME-MD diagnoses, whether they discussed PRIME-MD with their ED physician, whether their ED physician addressed psychiatric issues during their visit, whether they had been given a referral regarding psychiatric issues, and what types of health care they had received subsequent to the ED visit. At the end of the interview, the social worker attempted to help patients who desired intervention but had not received it find an appropriate source of care.

## Results

### Physician questionnaire

19 of 44 (43%) physicians whose patient did not have a PRIME-MD completed the short questionnaire. 3 of the 4 physicians in the tell-both group knew that the patient did not have a PRIME-MD psychiatric diagnosis. In none of the other 15 cases did the physician know the patient's PRIME-MD status.

20 of 51 (39%) physicians who treated patients with a PRIME-MD diagnosis completed the long-form questionnaire. In 10 of the 20 cases, the physician knew the patient's PRIME-MD diagnosis, including 7 of 8 cases in the tell-both group, 3 of 9 physicians in the tell-patient group, and 0 of 3 patients in the tell-neither group. The physician agreed with all of the PRIME-MD diagnoses in 5 of these cases, agreed with some in 4, and was unsure in 1.

6 of the 10 physicians unaware of the patient's PRIME-MD diagnoses agreed with all (2) or some (4) of them when informed of them. 1 disagreed, 2 had no opinion, and 1 did not answer. Only 2 of these 10 physicians wished that they had known the PRIME-MD diagnoses prior to beginning the patient interview.

6 physicians stated that they informed the patient about a psychiatric diagnosis (4) or wrote one in the medical record (4). The physicians who did not cited reasons such as "I was not confident of my diagnosis," "I did not want to antagonize my patient," "I did not want to stigmatize the patient," and "that was not what this visit was about." 2 physicians stated that they referred patients for further evaluation and treatment of their psychiatric condition. Those who did not cited "Did not believe patient would benefit," "ED not the place for this activity," and "patient did not meet DSM criteria for major depression," in addition to the aforementioned reasons.

### Patient questionnaire

Despite confirming phone numbers at the time of behaviour, and making up to 10 attempts at contact, we reached only 28 of the 51 patients with a PRIME-MD diagnosis. Most patients reported that their physical health and mental health were somewhat or greatly improved. 1 patient reported that her physical health had worsened. 1 patient reported that his mental health had deteriorated. Both of these patients had been provided with psychiatric referrals but neither had received care.

14 of 21 patients who were told their PRIME-MD status in the ED remembered it at the time of the call. Of these 14 patients, all but one correctly stated whether or not they had PRIME-MD diagnoses, although they were somewhat confused about which diagnoses they had, particularly with regards to the presence of mood or anxiety disorders. 5 patients (none from the control group) reported discussing their PRIME-MD status with their ED physician. 2 reported seeing a mental health specialist subsequent to the ED visit.

12 patients found the PRIME-MD evaluation "somewhat" or "very" helpful, 2 found it "somewhat harmful" and 14 were neutral. 22 were glad that they took it. None of the 6 who were not glad had a PRIME-MD diagnosis. Patients were generally grateful for the call and were generally not that concerned that their mental health needs (as identified by PRIME-MD) had not been met.

## Pre-publication history

The pre-publication history for this paper can be accessed here:



## References

[B1] Marchesi C, Brusamonti E, Borghi C, Giannini A, Di Ruvo R, Minneo F, Quarantelli C, Maggini C (2004). Anxiety and depressive disorders in an emergency department ward of a general hospital: a control study. Emerg Med J.

[B2] Gold I, Baraff LJ (1989). Psychiatric screening in the emergency department: its effect on physician behaviour. Ann Emerg Med.

[B3] Wulsin LR, Arnold LM, Hillard JR (1991). Axis I disorders in ER patients with atypical chest pain. Int J Psychiatry Med.

[B4] Schriger DL, Gibbons PS, Langone CA, Lee S, Altshuler LL (2001). Enabling the diagnosis of occult psychiatric illness in the emergency department: a randomized, controlled trial of the computerized, self-administered PRIME-MD diagnostic system. Ann Emerg Med.

[B5] Zane RD, McAfee AT, Sherburne S, Billeter G, Barsky A (2003). Panic disorder and emergency services utilization. Acad Emerg Med.

[B6] Byrne M, Murphy AW, Plunkett PK, McGee HM, Murray A, Bury G (2003). Frequent attenders to an emergency department: a study of primary health care use, medical profile, and psychosocial characteristics. Ann Emerg Med.

[B7] Katon WJ, Lin E, Russo J, Unutzer J (2003). Increased medical costs of a population-based sample of depressed elderly patients. Arch Gen Psychiatry.

[B8] Pfizer Incorporated (1997). PRIME-MD, version 12.

[B9] Spitzer RL, Williams JB, Kroenke K, Linzer M, deGruy FV, Hahn SR, Brody D, Johnson JG (1994). Utility of a new procedure for diagnosing mental disorders in primary care. The PRIME-MD 1000 study. JAMA.

[B10] Spitzer RL, Kroenke K, Williams JB (1999). Validation and utility of a self-report version of PRIME-MD: the PHQ primary care study. Primary Care Evaluation of Mental Disorders. Patient Health Questionnaire. JAMA.

[B11] Persoons P, Luyckx K, Desloovere C, Vandenberghe J, Fischler B, Persoons P, Luyckx K, Desloovere C, Vandenberghe J, Fischler B (2003). Anxiety and mood disorders in otorhinolaryngology outpatients presenting with dizziness: validation of the self-administered PRIME-MD Patient Health Questionnaire and epidemiology. Gen Hosp Psychiatry.

[B12] Leopold KA, Ahles TA, Walch S, Amdur RJ, Mott LA, Wiegand-Packard L, Oxman TE (1998). Prevalence of mood disorders and utility of the PRIME-MD in patients undergoing radiation therapy. Int J Radiat Oncol Biol Phys.

[B13] Rollman BL, Hanusa BH, Lowe HJ, Gilbert T, Kapoor WN, Schulberg HC (2002). A randomized trial using computerized decision support to improve treatment of major depression in primary care. J Gen Intern Med.

[B14] Schriger DL (2002). Problems with current methods of data analysis and reporting, and suggestions for moving beyond incorrect ritual. Eur J Emerg Med.

[B15] Spiegelhalter DJ, Abrams KR, Myles JP (2004). Bayesian Approaches to Clinical Trials and Health-Care Evaluation.

[B16] Eddy DM, Hasselblad V (1992). Fast*Pro. Software for Meta-Analysis by the Confidence Profile Method.

